# Endogenous Opioid Imbalance as a Potential Factor Involved in the Pathogenesis of Chronic Kidney Disease-Associated Pruritus in Dialysis Patients

**DOI:** 10.3390/jcm12072474

**Published:** 2023-03-24

**Authors:** Kamila Wala-Zielińska, Karolina Świerczyńska-Mróz, Piotr K. Krajewski, Danuta Nowicka-Suszko, Magdalena Krajewska, Jacek C. Szepietowski

**Affiliations:** 1 Department of Dermatology, Venereology and Allergology, Wroclaw Medical University, 50-368 Wroclaw, Poland; kamila.wala.01@gmail.com (K.W.-Z.);; 2 Department of Nephrology and Transplantation Medicine, Wroclaw Medical University, 50-556 Wroclaw, Poland

**Keywords:** pruritus, chronic itch, enkephalins, dynorphins, endorphins, chronic kidney disease-associated pruritus, chronic kidney disease

## Abstract

Chronic pruritus is one of the most common symptoms of dermatological diseases. It may occur in the course of other disorders, such as kidney disease. Chronic kidney disease-associated pruritus (CKD-aP) most often affects people with end-stage renal disease. The etiology of this condition is still not fully understood, but researchers are currently focusing on a thorough analysis of the association between disturbed opioid balance and increased neuronal signaling leading to pruritus. The aim of this study is to assess the concentration of endogenous opioids in dialysis patients with and without pruritus and in the control group, and to determine the correlation between the concentration of these substances and the occurrence and severity of itching. The study involved 126 dialysis patients and 50 healthy controls. Patients were divided into groups with pruritus (n = 62) and without pruritus (n = 64). The severity of pruritus was assessed using the NRS scale. The concentration of endogenous opioids was determined using the ELISA. The concentration of met-enkephalin was higher in the group of patients with pruritus compared to the control group. Moreover, significantly lower levels of β-endorphin and dynorphin A were observed in the group of dialysis patients compared to the control group. In addition, a statistically significant difference was seen between the β-endorphin concentration in the group of dialysis patients with pruritus compared to the group without pruritus. The ratio of β-endorphin/dynorphin A concentrations was significantly lower in the group of patients with pruritus compared to patients without pruritus and the control group. No correlations were found between serum level of studied opioids and the severity of pruritus. The concentrations of the studied opioids did not correlate with the severity of pruritus. Observed opioid imbalance may affect the occurrence of CKD-aP in dialysis patients, but a thorough understanding of the mechanism of action of these substances in the sensation of pruritus is necessary to assess the possibility of finding a new therapeutic target.

## 1. Introduction

Chronic itch (CI) is one of the most common symptoms of dermatological diseases and leads to significant impairment of daily functioning of patients [1]. CI may also occur in the course of other systemic conditions such as pregnancy, kidney and liver diseases, and lymphomas [2,3]. In chronic kidney disease, the incidence of CI, referred to as chronic kidney disease-associated pruritus (CKD-aP), depends on the severity of the disease and most often affects patients with end-stage renal disease requiring dialysis [4,5].

Importantly, it is emphasized that even half of patients with CI do not report their problem to a doctor. In addition, despite the use of currently available treatment methods, some patients do not respond to therapy at all or do not achieve sufficient improvement [6]. This leads to a significant reduction in the quality of life, an increase in patient mortality, and disturbances in everyday functioning, which may also lead to socio-economic consequences [7]. Luigi Naldi et al. [6] draws attention to the need to educate patients and create interdisciplinary pruritus treatment clinics that provide CI treatment with psychosocial support and innovative therapeutic methods.

Although the etiology of CI remains unclear, many pruritic mediators have been described that may play an important role in the pathogenesis of pruritus [1,8]. In addition, an attempt was made to identify clinical factors that can be positively correlated with the occurrence and increased severity of pruritus. For instance, G. Damiani et al. [9] reported that in patients with psoriasis, in which CI is present in up to 64–98% of cases, factors such as female gender, psoriasis of the head, face and genital area, pustular psoriasis, or lack of previous treatment are associated with higher itch intensity. Recently, many researchers have focused on a thorough analysis of the association between disturbed opioid balance and increased neuronal signaling leading to itching sensation [10,11,12]. The significant role of opioids in the pathogenesis of pruritus is suggested by the occurrence of itching in patients treated with opioids. This is particularly evident with epidural and intrathecal administration [12,13]. In order to analyze the influence of opioid disorders on the pathogenesis of CI, our current knowledge about the opioid system should be considered. So far, three main opioid receptors have been precisely identified and described, the activation of which will lead to specific, diverse changes in neuronal signaling. These are the δ-opioid receptor (DOR), μ-opioid receptor (MOR) and κ-opioid receptor (KOR) [14]. These transmembrane G protein receptors have been shown to be present in the cells of the peripheral and central nervous systems but are also expressed in keratinocytes, fibroblasts, dermal nerve ending, cells of the immune system, and secretory organs such as the pituitary [15,16]. In addition to the transmission of pain and itch stimuli, endogenous opioids are also responsible for modulation of the immune and cardiovascular systems, drug tolerance occurrence and, through the reward center, affect behavioral domain such as addiction and mood regulation [17,18]. Each of the opioid receptors is subject to the physiological influence of endogenous substances, which leads to the activation or inhibition of signaling. It should be emphasized that endogenous agonists are not fully specific for one receptor and also act on other receptors, but to a lesser extent, leading to a complex clinical response. DOR is activated mainly by endogenous enkephalins, MOR by endorphins, and KOR by dynorphins (A and B) [15,19].

Furthermore, in experimental studies, administration of MOR agonists has been noted to cause scratching in mice. In turn, the use of KOR agonists or MOR antagonists suppresses these reflexes [20,21]. These results led to the conclusion that MOR-dependent stimulation causes pruritus and that KOR-dependent signaling inhibits pruritus. The literature emphasizes the role of KOR and MOR in the pathogenesis of itching. However, recent research suggests that DOR can be involved in the modulation of pruritus at the spinal cord level [22]. In the study of Smith et al. [22] intrathecal administration of DOR agonists reduced chloroquine-induced itching in mice, while DOR antagonists stimulated pruritus. Numerous studies using endogenous opioids also supported previous observations. For example, endorphins, affecting mainly the MOR, cause an analgesic effect and depression of the respiratory system. With morphine-like properties, endorphins have been shown to cause pruritus and increase histamine-induced itching [23]. On the contrary, the administration of dynorphins, in addition to an analgesic effect, alleviates the itching via KOR [24]. Met-enkephalin and leu-enkephalin show the greatest affinity to the DOR and to a slightly less extent to MOR [25,26]. This may explain their main analgesic effect with less influence on pruritus [26]. It was also noted that the role of endogenous opioids in the formation and modulation of pruritus occurs through receptors present in the skin at the spinal cord level and in the central nervous system [27].

In pathological conditions such as neurological diseases (Parkinson’s disease, Alzheimer’s disease, spinal cord injury, epilepsy) altered opioid system activity has been noted [28]. A disturbed balance between the components of the opioid system has also been observed in dermatological diseases, including atopic dermatitis and psoriasis, which are often accompanied by pruritus [16,29,30]. The literature also suggests that alteration in opioid transmission may be important in the pathophysiology of CKD-aP. However, the number of clinical studies is insufficient to fully explain the mechanism of these disorders. It is assumed that, as in the case of other pruritic conditions, in CKD-aP there is an excessive activation of mu-opioid conduction with a decrease in KOR-dependent impulsation [5]. Therefore, in addition to determining the concentrations of individual substances, the ratio of the concentration of the MOR agonist (β-endorphin) to the KOR agonist (dynorphin A) is often calculated. The changed MOR/KOR ligand ratio indicates an increased activity and predominance of one of the elements of the opioid system [31]. However, the theory of an important role of opioids is mainly based on observations from clinical data demonstrating the efficacy of treatment of CKD-aP with KOR agonists and MOR antagonists. So far, there are only a few studies evaluating endogenous opioid concentrations and one study assessing the expression of opioid receptors in patients with end-stage renal disease [32,33,34,35].

Many patients with CI do not respond to standard therapy for pruritus, including antihistamines. Therefore, new therapeutic options for the treatment of CI of various origins are constantly sought [36]. Recently, aprepitant, a selective β/neurokinin 1 receptor antagonist, has been shown to be effective and significantly superior to antihistamines in the treatment of pruritus in patients with psoriasis [37]. Furthermore, clinical observations and animal studies have led to the discovery of new options for the treatment of CKD-aP with the use of opioid agonists or antagonists. For example, KOR agonists such as nalfurafine have been shown to reduce the severity of pruritus when administered to patients with CKD-aP [38]. KOR agonist—selective and peripherally acting difelikefalin is the first drug to have been approved by the FDA and EMA for the treatment of CKD-aP [39]. However, some dialysis patients do not respond to available therapies or their improvement is not sufficient. Therefore, taking into consideration the not fully explained pathogenesis of CKD-aP and the role of opioids in this disease, this area should be further explored in order to obtain new therapeutic options.

The aim of this study is to verify whether the concentration of endogenous opioids (Met-enkephalin, Leu-enkephalin, β-endorphin, and Dynorphin A) in the serum changes in dialysis patients with pruritus compared to dialysis patients without pruritus and a group of healthy people. In addition, it was also assessed whether there are correlations between the concentrations of these substances and the severity of pruritus.

## 2. Materials and Methods

### 2.1. Participants and Study Design

The study was conducted on a group of 126 patients from the Dialysis Unit at the University Hospital in Opole, Poland and the Department of Nephrology and Transplantation Medicine at the University Hospital in Wroclaw, Poland as well as 50 healthy subjects. All patients provided written, informed consent to participate in the study. Ethical approval was obtained from the Wroclaw Medical University Ethics Committee (Consent no. 26/2021, date: 29 January 2021). The study enrolled patients over 18 years of age who had been on dialysis 2 or 3 times a week for at least 3 months. Patients suffering from other diseases that may cause chronic pruritus, with psychiatric disorders and using antipruritic therapy were excluded from the study. Blood samples and basic demographic and clinical and data (age, gender, cause of CKD, duration of dialysis, type of vascular access) were collected from participants between November 2020 and April 2021.

### 2.2. Laboratory Tests

Initially, 9 mL blood samples were collected from 126 dialysis patients approximately 5–10 min before the next dialysis session and from 50 healthy study participants. Then, all samples (n = 176) were centrifuged for 15 min at 3000 rpm. Until further tests were performed, the research material was stored at −80 °C. The serum samples were distributed in 96-well plates and then, according to the manufacturer’s protocol, the enzyme immunoassay was performed. ELISA kits were used to evaluate the concentrations of met-enkephalin (S-1419 Met-Enkephalin ELISA Kit, BMA BIOMEDICALS, Augst, Switzerland), leu-enkephalin (Leu-Enkephalin EIA Kit, Phoenix Pharmaceuticals, Inc., Burlingame, CA, USA), β-endorphin (Nori^®^ Human β-endorphin ELISA Kit, GR111460-1, Genorise Scientific, Inc., Pennsylvania, PA, USA) and dynorphin A (RayBio^®^ Human Dynorphin A EIA Kit, Ray Biotech, Inc., Peachtree Corners, GA, USA), respectively. An EPOCH multiplate reader (BioTEK^®^ Instruments, Inc., Winooski, VT, USA) was then used to measure the absorbance at 450 nm.

Concentration of met-enkefalin, leu-enkefalin, and dynorphin A in serum were expressed in ng/mL and beta-endorphin in pg/mL. The ratio of MOR agonists to KOR agonists was calculated and presented as the ratio of plasma beta-endorphin concentration to dynorphin A concentration.

### 2.3. Pruritus Assessment

The severity of pruritus in CKD-aP patients was assessed using the numerical rating scale (NRS). Participants rated the worst severity of itching over the last 3 days on a scale of 1 to 10 (10 being the worst itch imaginable). The scores were then grouped by severity of pruritus into mild pruritus (NRS 1–3 points), moderate pruritus (NRS 3–6 points), severe pruritus (NRS 7–9 points), and very severe pruritus (NRS 9–10 points) [40].

Patients were also asked to complete the Polish version of the UP-Dial questionnaire [41]. It is a validated scale that has been developed to assess the severity of pruritus and its impact on quality of life specifically in CKD-aP dialysis patients. Components such as the severity, frequency and distribution of pruritus, skin changes caused by pruritus, as well as the impact on the patient’s sleep quality and psychosocial life are evaluated [41].

Moreover, the ItchyQoL questionnaire, a reliable, validated and designed for patients with CI instrument, was used to assess the quality of life of our patients. This questionnaire consists of 22 items (each item is scored from 1 to 5) evaluating 3 main domains: symptoms, functional limitation and emotions [42].

### 2.4. Statistical Analysis

All results were statistically analyzed using IBM SPSS Statistics v. 26.0 (SPSS Inc., Chicago, IL, USA). Initially, all the data were assessed for normal distribution. Qualitative results were subsequently assessed using the chi-square test. The T-Student and Mann-Whitney U tests were used for qualitative normally and abnormally distributed data. Pearson or Spearman correlations were used to analyze quantitative data based on normality. The differences in serum concentrations of the tested opioids between groups were analyzed using the Kruskal-Wallis test with the Bonferroni correction. All data are presented as mean ± SD. *p* < 0.05 was considered statistically significant.

## 3. Results

### 3.1. Characteristics of the Study Group

Among all participants, there were 91 (51.7%) women and 85 (48.3%) men. The mean age of the subjects was 63.9 ± 15.6 years in the non-pruritic patients, 61.1 ± 15.9 years in the pruritic patients, and 48.0 ± 10.2 years in the control group, respectively.

Patients underwent hemodialysis using arteriovenous fistulas (n = 82, 65.1%) or a tunneled internal jugular venous catheter (n = 44, 34.9%). In the group of patients with pruritus, patients with arteriovenous fistulas accounted for 51.6% (n = 32), patients with a central venous catheter—48.4% (n = 30). In the group without pruritus, 78.1% (n = 50) of patients had arteriovenous fistulas, 21.9% (n = 14) were patients with a central venous catheter. The difference in dialysis access was statistically significant (*p* < 0.005).

The mean duration of dialysis was 48.8 ± 51.9 months, 46.3 ± 58.4 months in patients without CKD-aP, and 51.4 ± 44.5 months in the group with CKD-aP, respectively. The most common causes of renal failure in all groups were diabetic nephropathy, glomerulonephritis, and ischemic nephropathy. Both the duration of dialysis therapy and the cause of renal failure are not significantly different in both groups of dialysis patients.

### 3.2. Serum Levels of Met-enkephalin, Leu-enkephalin, β-endorphin and Dynorphin A in Patients and in Control Group

**Figure 1:**
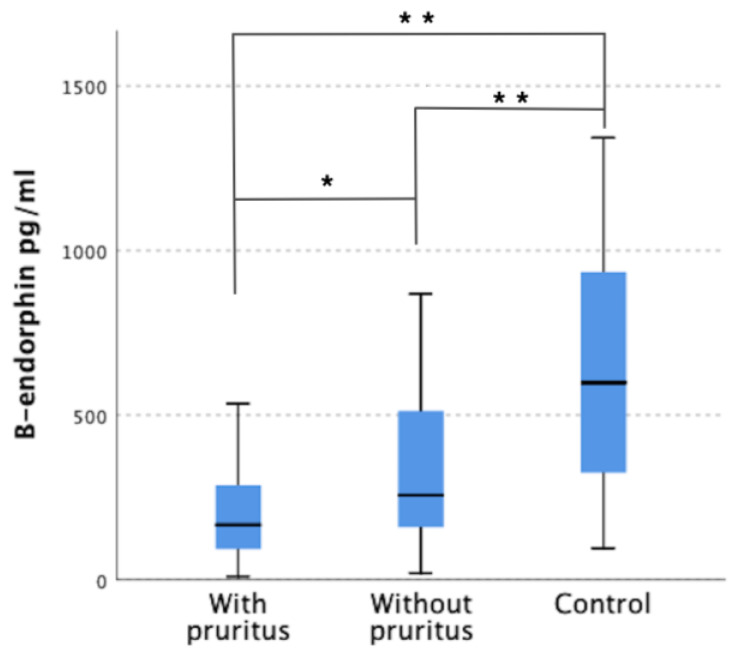
Serum level of β-endorphin in patients with pruritus, without pruritus, and control group. * *p* = 0.005; ** *p* < 0.001.

Significantly lower β-endorphin concentrations (*p* < 0.001) were observed in dialysis patients compared to the control group and were 216.25 ± 171.21 pg/mL in patients with pruritus, 344.84 ± 268.3 pg/mL in patients without pruritus and 658.51 ± 377.66 pg/mL in control group. In addition, a statistically significant difference (*p* = 0.005) was observed between the concentration of β-endorphin in the group of dialysis patients with pruritus compared to the group without pruritus (Figure 1).

**Figure 2:**
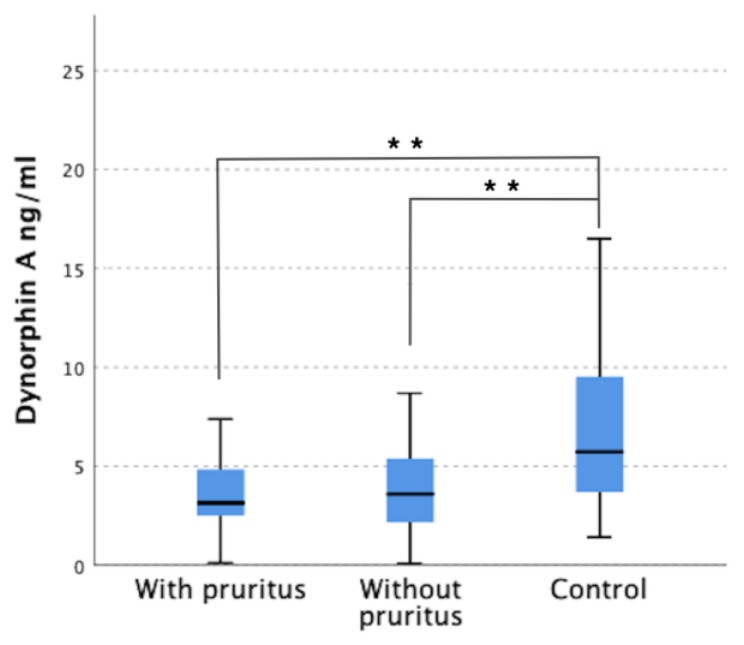
Serum level of Dynorphin A in patients with pruritus, without pruritus, and control group. ** *p* < 0.001.

Similarly, serum level of dynorphin A was lower in both groups of dialysis patients compared to control group (*p* < 0.001). However, no significant difference in dynorphin A concentrations was shown between group with and without pruritus, which were 4.32 ± 3.95 ng/mL and 4.62 ± 3.87 ng/mL, respectively (Figure 2).

**Figure 3:**
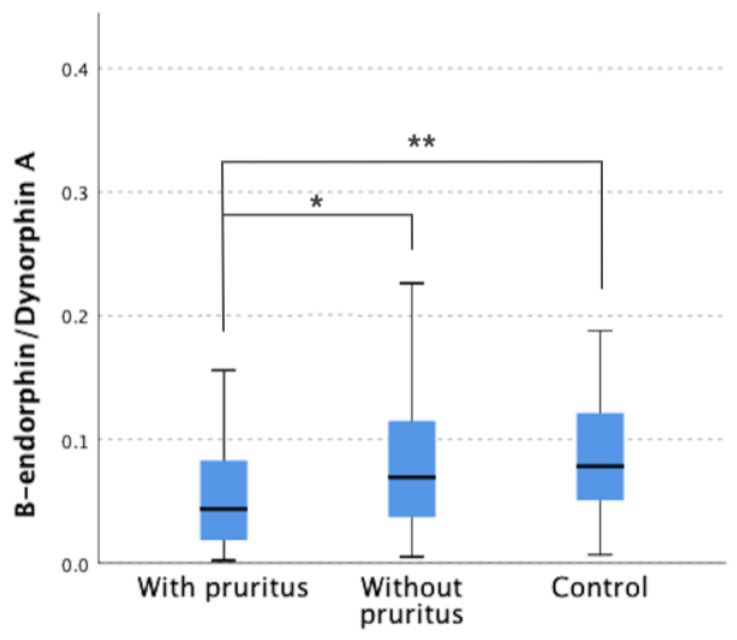
β-endorphin /Dynorphin A concentrations ratio in all study groups. * *p* < 0.05; ** *p* = 0.005.

Moreover, the ratio of β-endorphin to dynorphin A concentrations was 0.16 ± 0.64 in the group of patients with pruritus and was significantly lower compared to patients without pruritus (*p* = 0.048) and the control group (*p* = 0.005) (Figure 3).

**Figure 4:**
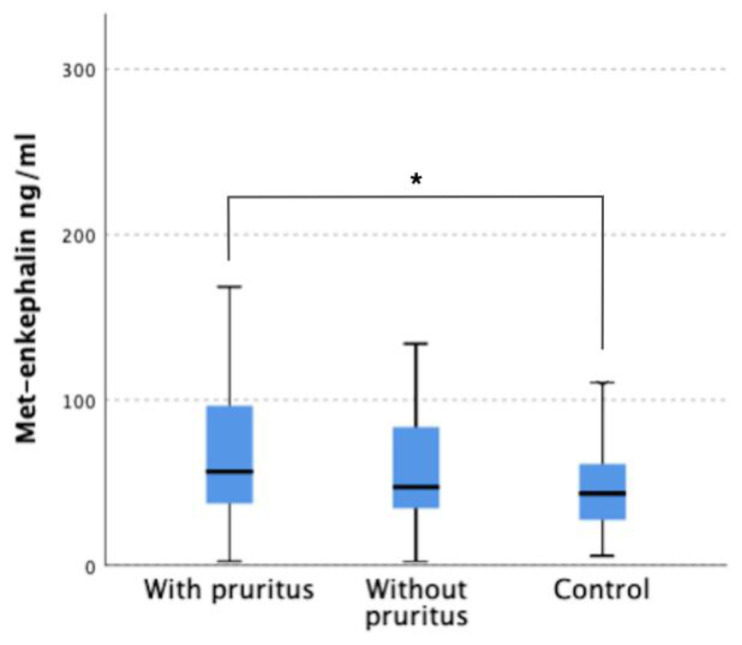
Serum level of Met-enkephalin in patients with pruritus, without pruritus, and control group. * *p* = 0.009.

**Figure 5:**
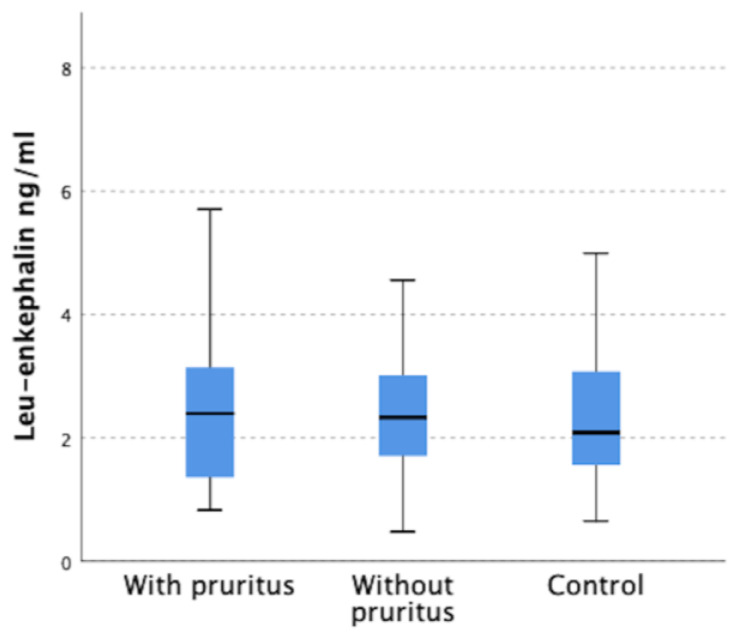
Leu-enkephalin concentrations in all study groups.

In turn, in the group of patients with pruritus the concentration of met-enkephalin was significantly higher compared to the control group (75.98 ± 65.48 ng/mL versus 48.06 ± 92.09 ng/mL) (*p* = 0.009). The concentration of met-enkephalin in pruritis patients also differed compared to the group without pruritus (61.37 ± 44.92 ng/mL), but in this case no statistically significant difference was obtained (Figure 4). There were no significant differences in leu-enkephalin serum levels between the study groups (Figure 5). Table 1 summarizes the results of opioid concentrations in the serum of study participants.

### 3.3. Correlations of Opioids Serum Concentration with Itch Intensity and Other Clinical and Demographic Data

The mean pruritic NRS score in dialysis patients with CI was 4.9 ± 2.2 points. Mild pruritus was reported by 16 (25.8%) CKD-aP patients, moderate pruritus by 30 (48.4%) and severe pruritus by 14 (22.6%) patients. Very severe pruritus (NRS 9–10 points) was observed in 2 (3.2%) patients. The concentrations of met-enkephalin, leu-enkephalin, B-endorphin and dynorphin A did not correlate with the severity of pruritus. The quality of life assessed by the participants (ItchyQoL scale) was on average 36.7 ± 13.7 points. Mean Up-dial total score was 14.2 ± 9.8 points. The concentration of the tested opioids did not correlate with the ItchyQoL score and the Up-Dial score.

The concentration of dynorphin A differed significantly in dialysis patients depending on the dialysis access and was lower in patients with a venous catheter (*p* = 0.026). In the case of other opioids, no similar correlations were observed. There were also no significant differences in serum concentrations of all tested opioids depending on data such as age, duration of dialysis and cause of renal failure.

## 4. Discussion

Undoubtedly, changes in the opioid system are involved in the pathogenesis of CKD-aP. This claim is supported both by the results of studies evaluating the concentration of endogenous opioids in CKD patients and clinical trials evaluating the effectiveness of drugs acting on opioid receptors, such as KOR agonists—difelikefalin, in reducing pruritus [14,31,35,38,43,44]. It is still not fully understood how opioid disturbances may affect the onset of pruritus in dialysis patients. The literature data published so far are inconsistent and refer to patients with pruritus caused by various diseases, such as cholestasis, psoriasis or atopic dermatitis [16,29,30,45,46,47].

According to Komiya et al. [48], an imbalance in the opioid system is one of the important factors in the pathogenesis of psoriatic pruritus. Clinical studies have shown a significant reduction in dynorphin A levels in psoriatic patients who complained of itching. However, in the mu-opioid system, both B-endorphin concentration and MOR expression remained unchanged in psoriasis patients compared to controls [48]. In contrast, other studies have shown elevated B-endorphin levels in inflammatory dermatological conditions such as atopic dermatitis, psoriasis and systemic sclerosis [49,50]. Furthermore, in experimental studies on mouse models, it was observed that in mice with imiquimod-induced psoriasis-like dermatitis and in atopic dermatitis Japanese mice, the administration of a MOR antagonist and a KOR agonist leads to the reduction of scratching, which additionally confirms the role of opioids in the sensation of itching in inflammatory dermatoses [51,52].

In our study, significantly lower B-endorphin and dynorphin A concentrations were obtained in dialysis patients compared to controls. However, only in the case of B-endorphin was a significant difference observed between pruritic and non-pruritic patients, with lower concentrations in the pruritic group. As studies emphasize the role of opioid balance in the pathogenesis of pruritus (and not just concentrations alone), the ratio of MOR agonists to KOR agonists is often evaluated. Such agonists include B-endorphin and dynorphin A, respectively. We have shown that this ratio is significantly lower in pruritic patients compared to non-pruritic patients and controls. In the study by Moniaga et al. [53] patients with chronic liver disease showed a lower concentration of dynorphin A but higher concentrations of β-endorphin in the group with cholestatic pruritus compared to the group without pruritus. In addition, a significantly higher β-endorphin/dynorphin A ratio was found in the pruritic group compared to non-pruritic patients and this ratio positively correlated with the severity of pruritus on the VAS scale, which is the complete opposite of our results [53]. In turn, in a study of Düll et al. [54] in patients with hepatic pruritus, a decreased concentration of dynorphin A was observed compared to patients without pruritus, however, the concentration of β-endorphin was also reduced, and thus no significant differences in the ratio of β-endorphin to dynorphin A concentrations between the study groups were observed [54]. To date, there have been only a few studies evaluating endorphin levels in CKD patients. Moreover, they were carried out in the last century. In a study conducted in the 1980s by Hwang et al. [33] dialysis patients showed elevated B-endorphin levels compared to healthy participants [33]. However, in a subsequent study in dialysis patients, in which the subjects were divided into pruritic and non-pruritic groups, no statistically significant differences in the concentration of B-endorphin were observed. In addition, no correlation was found between the serum concentration of this opioid and the severity of pruritus [34].

A little more research can be found on the concentration of enkephalins in patients with kidney diseases, including those on dialysis. Studies conducted in hemodialysis patients show that the concentration of met-enkephalin is higher in patients compared to healthy controls [55,56]. However, Danno et al. [56] reported no significant differences between the pruritic and non-pruritic groups. In turn, another clinical study showed a significant difference between patients undergoing dialysis with and without pruritus, however, it is worth noting that the study groups consisted of four patients each [35]. Data on leu-enkephalin levels in the serum of dialysis patients vary widely. The concentrations of this opioid described in the literature are both higher and lower in nephrological patients compared to the control group [55,57]. The studies did not assess the relationship between leu-enkephalin levels and the occurrence of pruritus [55,57]. In the case of hepatic pruritus, despite higher met-enkephalin and leu-enkephalin levels in patients with cholestatic diseases compared to controls, no statistically significant difference was observed between pruritic and non-pruritic patients [54,58].

Another important element in the pathogenesis of CKD-aP is the alteration in the expression of opioid receptors. A study in pruritic and non-pruritic dialysis patients assessed the expression of MOR and KOR in the skin and showed that KOR expression is downregulated in CKD-aP patients and correlates negatively with pruritus severity [32]. Similar results were obtained by Taneda et al. [59] in patients with pruritic psoriasis who had significantly reduced KOR levels in the epidermis compared to healthy controls [59]. Furthermore, in another study, biopsies of skin lesions in patients with psoriasis showed a reduced amount of KOR. A positive correlation was found between the downregulation of KOR expression and the severity of pruritus [60]. In all of these studies, there was no difference in the expression of MOR in keratinocytes among all study groups [32,59,60]. However, it should be emphasized that the modulation of pruritus also takes place at various levels of the nervous system (e.g., in the dorsal horns of the spinal cord) [24]. Receptor expression in the nervous system of CKD-aP patients has not yet been studied.

The results presented in this study do not provide a simple explanation for the pathogenesis of pruritus in CKD patients. It is worth noting that patients with liver disease or CKD have impaired metabolism or excretion of substances, which may lead to variations in the concentrations of the tested opioids [61]. For example, exogenous opioids such as morphine, oxycodone, and codeine accumulate in patients with renal impairment [62,63]. The increased effect of opioids may therefore not be due to increased synthesis but to impaired excretion. Therefore, it may be beneficial to measure opioid concentrations and also intensity of pruritus in patients both before and immediately after a dialysis session. Furthermore, the results obtained in this study raise the question of whether primary disturbances in the opioid system lead to pruritus or are secondary to pruritus, and the imbalance between KOR and MOR-mediated transmission in favor of KOR was intended to inhibit the impulses leading to itching. The differences in the concentrations of individual opioids in patients with CKD-aP demonstrated in the study, although they do not fully explain the pathogenesis of CKD-aP, confirm the role of the opioid system in the formation of pruritus in this group of patients. Therefore, it is reasonable to search for further therapeutic options focusing on the modulation of opioid transmission in the treatment of CKD-aP.

Our study is the first to evaluate the concentrations of multiple endogenous opioids (agonists of each of the three major receptors) in patients with CKD-aP. In addition, the study was conducted on the largest group of patients so far. However, the limitation of the study is the assessment of the concentration of endogenous opioids in the patient’s serum only before the dialysis session. It has not been evaluated whether these concentrations change after dialysis. In addition, expression of the opioid receptors (MOR, KOR and DOR) as well as production of opioids in the skin of patients was not assessed in this study.

## 5. Conclusions

Disturbances in the balance between the concentrations of the individual components of the opioid system may contribute to pruritus or modulate itching sensation in dialysis patients. Further studies, including determination of the expression of opioid receptors, may help researchers to understand the exact mechanism of action of these substances in the pathogenesis of CKD-aP.

## Figures and Tables

**Table 1:** Concentrations of Met-enkephalin, Leu-enkephalin, β-endorphin, and Dynorphin A in serum in the studied group.

Parameter	Group 1—With Pruritus		Group 2—Without Pruritus		Group 3—Controls		*p*-Value
	Mean ± SD	Median	Mean ± SD	Median	Mean ± SD	Median	
Met-enkephalin (ng/mL)	75.98 ± 65.48	58.6	61.37 ± 44.92	47.3	48.06 ± 92.09	43.3	0.033
Leu-enkephalin (ng/mL)	2.9 ± 2.15	2.5	2.79 ± 2.1	2.3	2.42 ± 1.22	2.1	NS
β-endorphin (pg/mL)	216.25 ± 171.21	166.4	344.84 ± 268.3	256.6	658.51 ± 377.66	596.2	<0.001
Dynorphin A (ng/mL)	4.32 ± 3.95	3.1	4.62 ± 3.87	3.6	8.1 ± 6.2	5.8	<0.001
β-endorphin/Dynorphin A	0.16 ± 0.64	0.047	0.16 ± 0.34	0.076	0.13 ± 0.14	0.086	0.004

NS—not statistically significant, SD—standard deviation.

## Data Availability

The datasets generated and analyzed in the current study are available from the corresponding author on reasonable request.
